# A Process Evaluation of a Program to Retain Clinical Scientists with Caregiving Responsibilities

**DOI:** 10.1177/26884844251389901

**Published:** 2025-10-24

**Authors:** Devin A. Madden, Jenny J. Lin, Timnit Berhane, Richa Deshpande, Carol R. Horowitz, Sandra K. Masur, Sasha Perez, Toni Stern

**Affiliations:** ^1^ Office of Career Opportunity and Support, Icahn School of Medicine at Mount Sinai, New York, New York, USA.; ^2^ Department of Population Health Science & Policy, Icahn School of Medicine at Mount Sinai, New York, New York, USA.; ^3^ Geriatrics and Palliative Medicine, Icahn School of Medicine at Mount Sinai, New York, New York, USA.; ^4^ Departments of Ophthalmology and Pharmacological Sciences, Icahn School of Medicine at Mount Sinai, New York, New York, USA.

**Keywords:** gender equity, academic medicine, caregivers in academic medicine, faculty support programs, process evaluation of faculty support programs

## Abstract

**Introduction:**

Caregiving responsibilities in academic science, technology, engineering, mathematics, and medicine disproportionately impact early-career faculty, particularly women, exacerbating stress, mental health challenges, and career progression barriers. The Doris Duke Charitable Foundation (DDCF) launched the Fund to Retain Clinical Scientists to support clinician-scientists facing these challenges, expanding efforts during the COVID-19 pandemic. The Icahn School of Medicine at Mount Sinai leveraged DDCF funding in 2021 to expand its Distinguished Scholar Award (DSA) program, first launched in 2020.

**Methods:**

To enhance faculty support, we convened an interdisciplinary team with expertise in faculty development, integrating tailored programming through collaborative working sessions. In 2022, seven scholars were funded; in 2023, five were awarded. The cohorts represented diverse gender identities, racial and ethnic backgrounds, and research disciplines.

**Results:**

Summative evaluations revealed that 83% of respondents found quarterly check-ins with program directors most useful, followed by “Meet the Expert” sessions (67%). Findings highlight the value of structured mentorship and institutional support in fostering research continuity and gender equity.

**Discussion:**

Over 2 years, the DSA program provided both financial and professional development support, addressing key challenges faced by junior faculty with caregiving responsibilities. While scholars benefited from mentorship and career development opportunities, participation in additional resources varied, raising questions about how best to balance program structure with flexibility. The ongoing challenge remains on how to equitably support caregivers in science and medicine without adding undue burdens. Our experience underscores the importance of continued dialogue and strategic program refinement to ensure lasting impact.

## Background/Introduction

In recent years, there has been increased attention on employees’ experiences with caregiving burden, highlighting how caregiving results in stress exacerbation and poorer mental health, such as worse anxiety and depression.^1,2^ Moreover, there are unique sets of challenges for caregivers based on career type. Within academic science, technology, engineering, mathematics, and medicine (STEMM), beyond stress and mental health issues, caregivers face challenges related to both career momentum and gender disparities. These challenges disproportionately affect early-career faculty, 70% of whom find themselves debating whether they can stay their career course.^3^ These concerns are also starker for women in STEMM, who disproportionately experience the negative career consequences associated with these caregiving role conflicts.^4^

As a response to specific challenges around maintaining research momentum while navigating caregiving in academic medicine, the Doris Duke Charitable Foundation (DDCF) launched The Fund to Retain Clinical Scientists (FRCS) in 2015. DDCF’s funding goal was to foster gender equity in academic medicine by supporting and thus retaining women scientists who bear the disproportionate burden of caregiving responsibilities.^5,6^ In 2020, Icahn School of Medicine at Mount Sinai’ (ISMMS) Office of Career Opportunity and Support (OCOS), formerly the Office of Gender Equity in Science and Medicine, drew inspiration from the FRCS and other programs across the country and launched the Distinguished Scholar Award (DSA) to support early career investigators in maintaining independent research careers while integrating caregiving responsibilities into their lives. The program was available to caregivers of any gender, a decision that aligned with DDCF’s goals of enhancing gender equity in academic medicine by acknowledging that people of all genders must take on caregiving responsibilities if gender equity is to be achieved.^4,7^ This focus helps counter biases against caregiving that have stemmed from these responsibilities being traditionally relegated to one gender.^7^

In 2021, DDCF announced the COVID-19 FRCS, recognizing how the onset of the COVID-19 pandemic disrupted the medical workforce in many ways. As context, COVID-19 brought with it physical, social, and emotional shifts to the job and work environment, producing noticeable changes in scientific research, publications, and productivity.^5,6,8^ These effects were particularly pronounced among women (students, trainees, scientists, and clinicians), who were nearly twice as likely as men to report experiencing caregiving-related exhaustion as a stressor.^5^ In addition, individuals from historically marginalized and underrepresented communities faced heightened challenges during this time.^5,6^ ISMMS, through OCOS, was one of 22 U.S.-based medical schools to receive two years of DDCF FRCS funding to support junior faculty with research careers as they navigated the unique landscape of academic medicine during the pandemic.

**FIG. 1.**
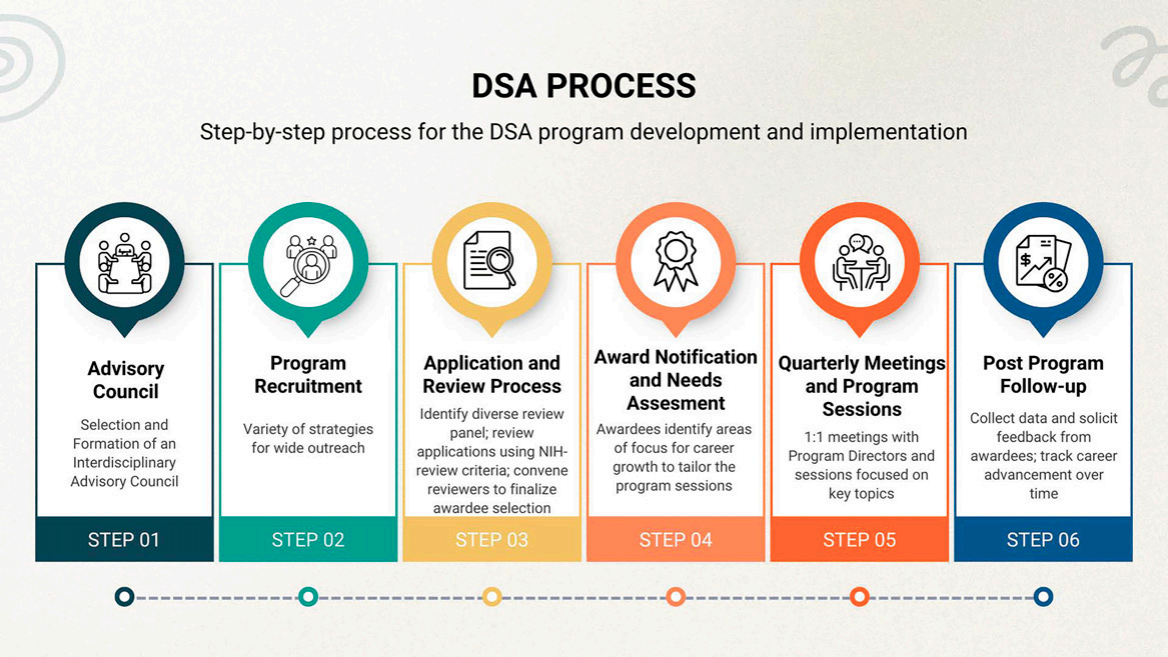
Visual representation of Distinguished Scholars Program process.

To better understand the impact of DDCF’s FRCS programs, researchers, to date, have conducted interviews with leaders and awarded recipients at funded sites^9,10^ and found that individuals perceived the program as needed, beneficial, and a clear signal that their institutions validated and supported their dual responsibilities as caregivers and scientists.^9^ While research on these programs has examined aggregated results across funded institutions, our work provides an opportunity to evaluate the process by which OCOS at ISMMS envisioned and implemented the DSA program with funds received from DDCF in 2022. The purpose of this paper is to share how we developed, implemented, and assessed impact for our DSA program. This includes demonstrating the programming needs we witnessed, how we sought to address those needs, and what we learned along the way, offering a potential roadmap for other institutions (see Fig. 1).

## Methods

### Program development

The funding opportunity from DDCF in 2021 provided OCOS the opportunity to expand the DSA program in the face of emerging needs unveiled by the pandemic. In addition to funding an increased number of eligible scholars directly, the DDCF FRCS award allowed for expansion of programmatic support.

**FIG. 2.**
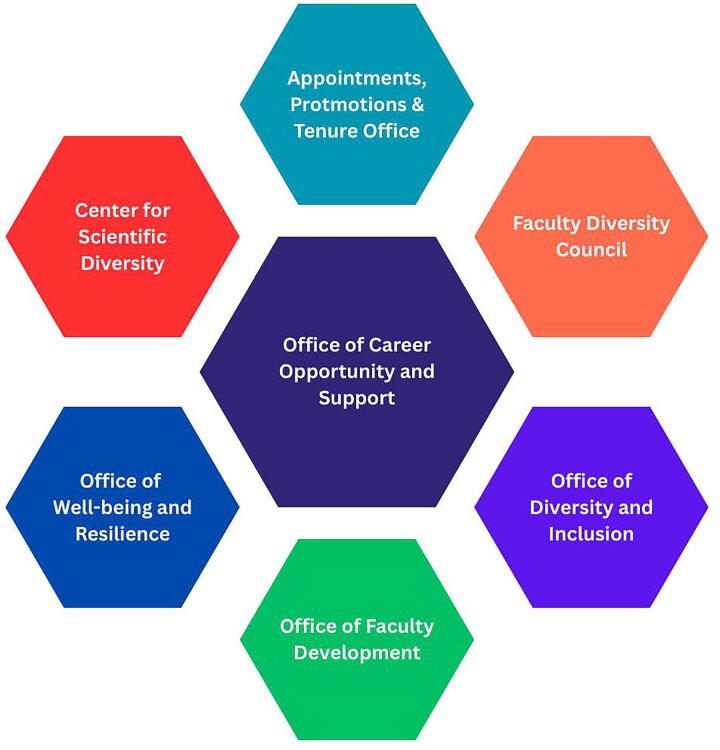
Offices represented on the Distinguished Scholars’ Program’s internal interdisciplinary advisory committee.

To inform the expansion of the DSA program, OCOS brought together an advisory council. The advisory council was an interdisciplinary team of individuals from internal partnering offices and councils with experience and expertise in faculty support and development (Fig. 2). Each partnering office has a robust portfolio supporting ISMMS faculty and thorough understanding of faculty development and the challenges facing early-career scientists. After initial planning meetings, leveraging a team science approach, we finalized the process for publicizing and recruiting potential applicants and structuring and formatting the program. The group continued to meet, in smaller groups and at quarterly group working meetings, to share relevant programming each office or council was offering, which we either adapted or offered to scholars through our DSA curriculum.

The advisory council also helped finalize the eligibility criteria and supported recruitment. Eligible faculty included clinical/translational or basic scientists at the Assistant Professor level or within their first year as an Associate Professor with active extramural support for their research programs. Applicants had to devote at least 50% of their effort to research. Awardees received $50,000 in funding to support their research. The award could be used for support of research personnel (*e.g.,* technician, research coordinator, data analyst), dissemination services (*e.g.,* media support, professional data visualization), career development activities (*e.g.,* professional coaching), or other research/career development expenses.

To recruit, OCOS prioritized program visibility and the capacity to reach a spectrum of potential applicants. We employed multiple channels to announce and promote the DSA program, leveraging our partners to share the DSA announcement through their networks to reach a diverse pool of potential applicants. We shared flyers and information through email listservs and provided partners across the school with a predesigned presentation slide deck to showcase during formal presentations, including Grand Rounds. The program was discussed at the ISMMS Dean’s meeting with Chairs and Institute Directors, and following this meeting, OCOS staff emailed materials to all Deans, Chairs, Institute Directors, and their administrators. Finally, to ensure that the information was accessible and clear, OCOS program leads offered an informational session by Zoom to answer questions about the application process and/or program. The session was recorded and made available for asynchronous viewing. The call for applications was open for 6 weeks in early-mid winter.

### Application and review process

In addition to demographic information (gender, race/ethnicity, disability, disadvantaged background) and a curriculum vitae, the application included sections on career development plans, research approach, statement of caregiving need, and budget justification. We required two letters of support, one from the primary research mentor and the second from a former mentor or a current chief or chair. Applicants were also asked to share what they perceived to be their top three areas of need for additional support. Applications were submitted *via* REDCap, a secure web-based database.^11^

Program directors (PDs) within OCOS identified potential senior faculty for the review committee based on their scientific understanding of the applicants’ research areas. All review committee members had to confirm that they did not have a conflict of interest and could provide an honest and comprehensive review. For this review, a conflict was defined as being a mentor, coauthor, or funded coinvestigator with the applicant in the prior 2 years. Prior to the review, committee members attested that they had completed antibias training.

Review committee members evaluated applications using a rubric and scoring scale that mirrored the National Institutes of Health (NIH) peer review selection criteria for K-award applicants. Scored criteria included: candidate (*e.g.,* potential as a researcher, appropriate training and experience, evidence of commitment to independence); career development plan/career goals and objectives; research plan; mentoring team; and overall impact. While caregiving needs were required, and particularly those linked to COVID-19 were considered, they were not scored due to concerns that quantifying such needs might introduce bias.

Each application was scored by two to three reviewers, and those applications with scores in the top half were discussed at a subcommittee review meeting, conducted as an abbreviated version of an NIH study section review meeting. Awardees were selected among the top-scored applications with the OCOS team also considering the diversity of science represented. All applicants received aggregate reviewer comments as feedback to further their career development.

Finally, review committee members also provided feedback on the review process and responded to a short quality improvement survey on the process’s transparency.

### Programming implementation

Awarded scholars were notified of their acceptance into the program *via* a signed letter from the Dean of ISMMS and PDs. Following notification, OCOS requested all awardees to complete a more comprehensive needs assessment to inform final curricular programming to be responsive to their needs. Based on the results of this needs assessment (discussed further below) and input from our Advisory Council, we built a calendar of programming and support that included leadership and research training opportunities, individual meetings with senior leaders, cohort gatherings with experts across the institution, cross-cohort networking, an online community, professional video development to enhance career visibility, and access to a hub of additional resources, including links to caregiving resources that were available through the institution (Table 1). In addition to programming and resources, PDs met with each scholar individually quarterly and discussed support with identifying additional mentors, navigating difficult conversations with mentors and other team members, obtaining direct feedback on research progress, managing research logistics (*e.g.,* study or team management), challenges and successes with balancing work with caregiving responsibilities, and academic promotional processes.

**Table 1.** Topics of Curricular Program by Domain

Career competency development	Coaching and networking	Science spotlights
Building a research career-workshops	Quarterly meetings with PDs	Professionally produced videos highlighting awardees’ research
Understanding IRB	One on one meeting with Medical School Dean (and pre-meeting prep)	System-wide broadcasts
Successful grant writing	Monthly “Meet the Experts” series featuring senior leaders from Health System	
Effective time management	Cohort networking and support	
Creating your elevator pitch	Cross-cohort networking and support	
Using social media for your research	Online community and web portal	
Managing budgets		
Building teams and leadership skills		
Team science		
Mentoring competency assessments		
Mentoring self-practice workshop resources		
Meyers Briggs Type indicator for optimizing team dynamics		

To assess the impact of our programming, we created a summative post-program evaluation. This included requests to all scholar cohorts to complete progress reports, a survey of the professional development provided, and attendance at quarterly check-in meetings. We also reviewed notes from the scholars’ individual meetings with senior leaders for insights.

## Results

### Recruitment—applicants and awardees

**FIG. 3.**
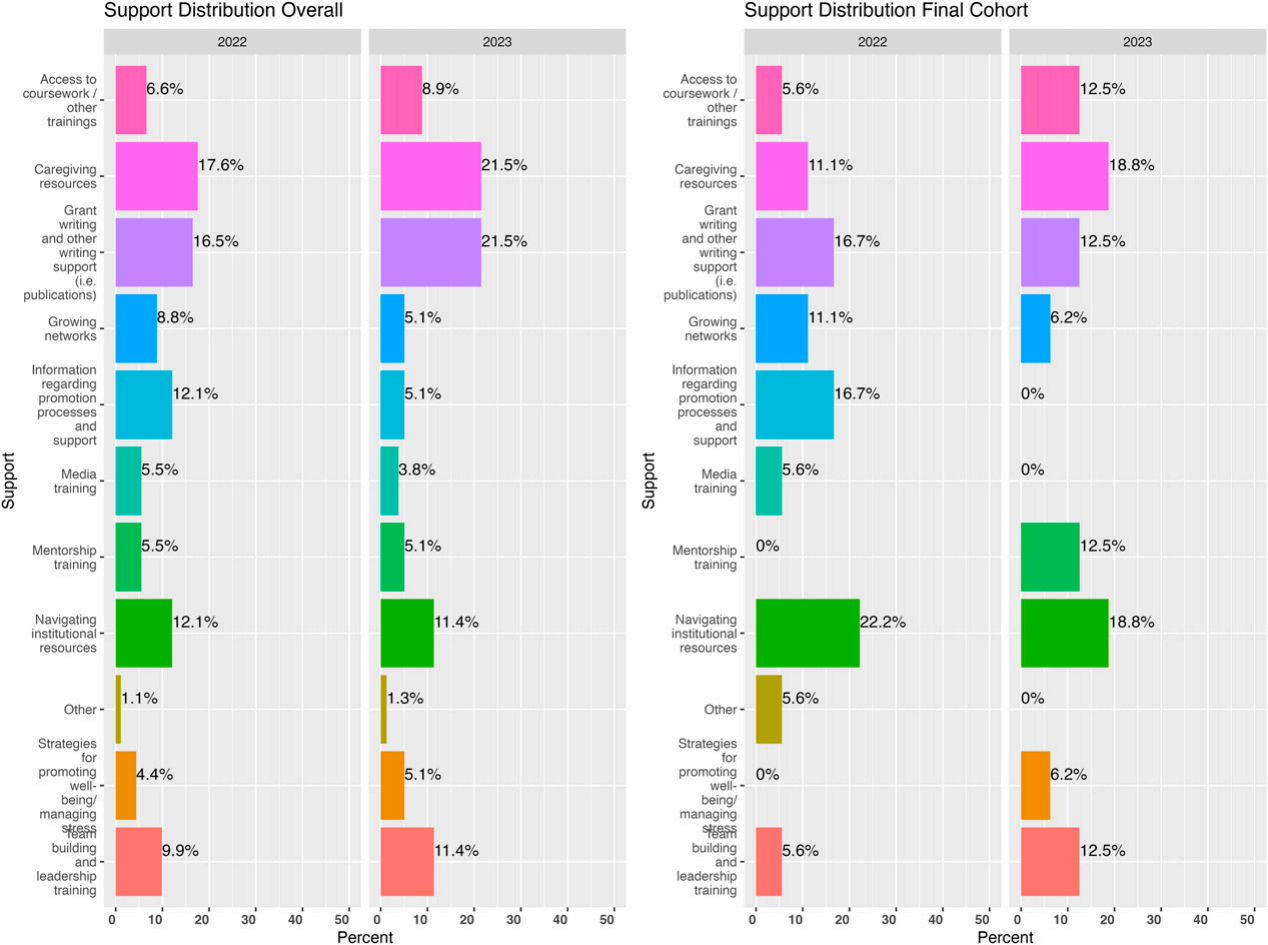
A visual comparison between awardees’ initial most noted needs and needs of the entire applicant pool.

Table 2 provides a demographic overview of the applicants and awardees by year, and Figure 3 highlights prioritized support areas and needs as identified by applicants in each cohort.

**Table 2.** Applicant Demographics

Characteristic	2022		2023	
	Applicants *N* = 29	Awardees *N* = 7	Applicants *N* = 28	Awardees *N* = 5
Gender, *N* (%)				
Cisgender woman	22 (76)	5 (71)	24 (86)	5 (100)
Cisgender man	5 (17)	1 (14)	2 (7)	—
Nonbinary person	2 (7)	1 (14)	1 (4)	—
Prefer to self-describe	—	—	1 (4)	—
Race/ethnicity, *N* (%)				
Asian	7 (23)	2 (29)	6 (21)	1 (20)
Black or African American	3 (10)	1 (14)	1 (4)	1 (20)
Hispanic or Latinx	2 (7)	1 (14)	3 (11)	—
White	18 (60)	3 (43)	15 (54)	3 (60)
Other	—	—	3 (11)	—
Current rank/appointment, *N* (%)				
Instructor	1 (4)	—	—	—
Assistant Professor	23 (79)	6 (86)	24 (86)	4 (80)
Associate Professor	5 (17)	1 (14)	4 (14)	1 (20)
Years at current rank, *N* (%)				
<1	13 (45)	3 (43)	11 (39)	3 (60)
1	3 (10)	1 (14)	5 (18)	1 (20)
2–6	11 (38)	3 (43)	12 (43)	1 (20)
>6	2 (7)	—	—	—
Concurrent funding, *N* (%)				
NIH	19 (66)	5 (71)	13 (46)	3 (60)
Intramural funding	14 (48)	3 (50)	16 (57)	3 (60)
Terminal degrees, *N* (%)				
MD/DO	10 (35)	5 (1)	13 (46)	3 (60)
PhD	19 (66)	2 (29)	15 (54)	2 (40)
Disabled, *N* (%)	1 (4)	—	1 (4)	—
Disadvantaged background, *N* (%)	3 (10)	—	5 (18)	1 (20)
Outreach types, *N* (%)				
Broadcast email	25 (42)	6 (40)	21 (45)	3 (38)
Chair or Division Chief	8 (13)	3 (20)	6 (13)	1 (13)
Listserv email	10 (17)	3 (20)	5 (11)	1 (13)
Mentor	8 (13)	3 (20)	11 (23)	3 (38)
Other	2 (3)	—	1 (2)	—
Other advisor	7 (12)	—	3 (6)	—

#### Applicants

We received 29 applications in 2022 and 28 applications in 2023. The demographics for each application cycle are shown in Table 2, including years at rank, concurrent funding, terminal degree, and mode of DSA outreach. Applicants’ research areas ranged from basic science to clinical/translational work in diverse fields such as asthma, cancer, depression, maternal health, spatial transcriptomics, health disparities, virology, long COVID, and structural racism, to name a few.

Applicants from both cohorts reported caregiving resources (20%) as their most pressing area of need, followed closely by grant writing and other writing supports (19%). Navigating institutional resources (12%) ranked as the third most frequently noted need.

#### Review process

Across both years, we had 25–28 reviewers per year participate in the process. The PDs recruited socio-demographically diverse reviewers who also represented applicants’ breadth of departments and fields. In qualitative feedback about review process, many reviewers shared that they found the review process to be transparent, including comments such as “*All voices were heard”* with a “*Clear emphasis placed on the key elements of candidacy.”* Some reviewers cited their uncertainty about the ways in which they should consider caregiving burden and noted that this made the process more difficult.

#### Awardees

In 2022, we awarded seven scholars. Of these, five identified as cisgender women, one as a cisgender man, and one as a nonbinary person. There was racial and ethnic diversity, with two scholars identifying as Asian, one as Black, one as Hispanic/Latine, and three as White. Most of the scholars had extraprofessional challenges related to caregiving for small children, while one of the scholars was caring for a sick parent. The scholars represented diverse departments, including Pediatrics, Oncological Sciences, Emergency Medicine, Infectious Disease, Women’s Health, and Population Health Science and Policy. Four were clinical researchers, and three were basic scientists. Four scholars held MDs, two had PhDs, and one held a DrPH. Several months into programming, one of the scholars relocated across the country to pursue another career opportunity, bringing this cohort to six scholars.

In 2023, five scholars were awarded funding. All identified as cisgender women; three identified as White, one as Asian, and one as Black. All scholars had extraprofessional challenges related to caregiving for children (across ages). Their research spanned the fields of geriatrics and palliative medicine, orthopedics, psychiatry, obstetrics, gynecology and reproductive science, diabetes, obesity and metabolism, and gastroenterology. Three were clinical researchers with MDs and two were basic scientists with PhDs.

#### Awardees’ programming needs

In both cohorts, developing and establishing a scholarly reputation was a predominant need, cited by a majority (four) of awardees. In 2022 to 2023, the majority (five of seven) in the cohort felt they needed grant/fellowship writing support; this was only cited as a need by two of the five scholars in the 2023 to 2024 cohort. In addition, four of seven scholars in the 2022 to 2023 cohort requested leadership skills support (*e.g.,* communication, empathy, decision-making, and team building), which was only ranked as a need by two scholars the following year. For the 2023 to 2024 cohort, the second highest need (for three of the five scholars) was in managing research logistics (*e.g.,* navigating IRB, working with grants and contracts offices).

Finally, when asked to summarize research or work-related barriers experienced during the pandemic that negatively impacted their ability to advance their careers, scholars listed competing demands, a lack of protected time for their own research, and frequent childcare interruptions or demands. They also noted difficulties with a more remote world resulting in reduced face-time with their mentors and sponsors, a lack of in-person networking, and difficulty managing teams or laboratories with remote schedules. We strove to address these barriers through this award, as discussed in the programming implementation noted in methods. Of note, during this time, we worked hard to establish backup childcare benefits that employees, including these scholars, could also leverage.

During quarterly meetings with PDs, scholars also shared requests for additional programming; these requests included workshops on Institutional Review Board management, grant writing, and leveraging social media for research. In some cases, scholars used the opportunity to discuss how bias shows up in their field. Some scholars also discussed that making the DSA programming a requirement would help ensure that they carved out time from their schedules to participate in the useful seminars. Scholars shared that the professional video was one of the most important and beneficial aspects of the program and that sharing the video led to improvements in their networking outreach.

#### Summative evaluations

Six of the 11 (55%) scholars responded to the evaluation to rank the three professional development offerings that were most useful to them. The majority (83%) ranked the quarterly check-in meetings with DSA PDs among the most useful, followed by the “Meet the Expert” sessions (67%). Three (50%) respondents indicated that the *Understanding Yourself and Others through the Myers-Briggs Type Indicator workshop* and individual meetings with the Dean were among the most useful.

When asked what they found to be most helpful about these professional development offerings, participants spoke about the importance of having designated time to commit to professional development. They also spoke to the importance of having opportunities to interact with other people, including presenters from the “Meet the Experts” series, and spoke to the positive ways that this contributed to both networking and mentorship.

When asked about future programs, participants wanted more time to meet with others from their cohort, including time for in-person sessions to facilitate collaboration. One participant noted the need for personal career coaching focused on short- and long-term career goals, and another discussed the need for practical skills programming on the topics of effective presentations, successful job interviewing techniques, and media training.

When asked specifically about the professional videos produced through this program for scholars to use to highlight their science and uplift their scholarly reputation (an area of need participants cited), one participant noted that it was, *“extremely helpful to show the public what I am doing and what my research program looks like […] I have used it for student recruitment, outreach programs, lab website, and have posted on social media.”*

Progress reports collected 1 and 2 years following the conclusion of their program indicate that the scholars maintained their productivity. Together, the 2022 cohort have published at least 19 papers related to the research funded in part by this program and over 50 papers since 2023. Five have received K awards from the NIH, and two have received R01 awards from the NIH since 2023. Three have been promoted to associate professor. The scholars from our 2023 cohort have collectively published at least 6 manuscripts related to their research and at least 19 papers since 2024. Three scholars have received NIH K awards since 2024, and one was awarded an NIH R01. Two have been promoted to associate professor.

All scholars, across cohorts, continue to apply for additional funding, including R01 awards. We will continue to track up to 5 years post-program conclusion and suspect promising outcomes based on these preliminary results.

## Discussion

Programs such as the Distinguished Scholar Award, including those supported by the DDCF FRCS, are critical to enhancing equity by supporting early investigators as they manage maintaining research momentum against navigating caregiving demands.

As demonstrated by the number of applications received both years from a range of disciplines and backgrounds, there is both an interest and need among early investigators for this kind of support. Leveraging multiple recruitment channels has been effective for reaching a broad range of candidates with diverse needs. While system-wide broadcasts proved to be most effective, it is important to consider ways we can better leverage mentors and department chairs in encouraging junior investigators to put themselves forward for awards such as this.

While it is difficult to attribute participation in this program directly to maintained productivity, when considered against the feedback the scholars provided to PDs and the success of similar programs in sustaining junior faculty,^12^ the continued success of our scholars is a promising sign that the support is making a difference. Conducting longer-term, follow-up qualitative program assessments that allow participants to reflect directly on the program’s influence on their career trajectory would contribute to our collective understanding and offer an opportunity to refine curricular offerings moving forward. With these needs in the forefront of future planning, this iterative and ongoing evaluation revealed several considerations for teams to best operationalize these programs to optimize quality and overall benefit for participating scholars.

Providing early career investigators with professional development opportunities tailored and responsive to their needs shows promise for long-term success and productivity^13,14^; such opportunities may be shelved for more pressing needs among researchers navigating emergent or ongoing caregiving challenges, particularly after the disruptions of the pandemic, and this award sought to provide them time, space, and resources to address that. Our programming team built optional professional development into the award with the belief that it would add value, and several scholars spoke to positive result of the program’s prioritization of time for professional learning. We also created an online Microsoft Teams page for each cohort to review materials asynchronously and chat with members of their cohort, as needed. We included updates, workshop presentations, and notification for each cohort within this Teams page. However, despite conducting needs assessments to understand and developing a programming calendar relevant to scholars, participation in workshops was inconsistent.

Furthermore, we had low completion rates of summative assessments, a common barrier to professional development programs, making it difficult for us to assess whether the content delivered resonated with the scholars. Continuing to offer professional development will require us to better understand why there was a lack of steady participation. Determining if this was the result of scheduling conflicts, a lack of interest in the topics offered, or the evolving demands of the scholars’ careers and their individual processes of prioritization for non-mandated activities will help us determine appropriate next steps. Finally, given this funding opportunity is meant to assuage the numerous competing demands on the scholars’ careers, mandating additional professional development may feel onerous and burdensome. However, in meetings with the PDs, several scholars reflected that required programming would be helpful. Finding the right balance between mandatory programming and honoring the many demands these scholars face will be an ongoing, important effort.

Finally, scoring caregiving responsibilities and needs is sensitive, contentious, and important to give more thought to. For the purposes of our review, scholars were not assessed based on the breadth of their need. While some reviewers felt this maintained the integrity of the program and allowed us to support caregivers with varying needs, others felt it detracted from the equity lens. Similar discussions have emerged among other institutions that have scored caregiving, with PDs observing that ranking need means those with caregiving challenges perceived as typical are less likely to receive the award and are left without institutional support programs like this are meant to, in part, rectify.^15^

## Conclusion

With support from the DDCF FRCS for 2 years, we sought to bolster junior faculty with caregiving demands during what could be a tenuous time for their career development. Through interdisciplinary efforts informed by the participants’ cited needs, we built cohort models of professional development programming that accompanied a monetary award granted to top applicants. Over the 2 years of the program, we had conversations with and collected data from both award reviewers and each of the scholars to learn where the program was most helpful and where to adjust. The need for similar programs is evident in the number of applications we received, the vast array of needs applicants and awardees cited in both needs assessments and their applications, and the frankness scholars offered in their conversations with PDs about the challenges they faced. Though the DDCF FRCS program has been discontinued, the need for this program, and its promise, has continued to draw interest from the school’s dean and funders. We continue to offer this award and consider how to best execute programming that responds to the needs of each unique cohort, allows for a cohort-building effect, is sensitive to the existing demands awardees face, and meets those with high needs to promote equity. It remains a challenge to consider.

The purpose of this paper is to demonstrate that there is a need, encourage institutions that similar programs can be successful in supporting scholars with such needs, and provide a potential implementation model, with lessons learned along the way, that institutions can consider as they build their own programs. Our experience demonstrates that awardees, all of whom have a caregiving need but whose need was not ranked, both value and benefit from the financial support and direct access to mentorship (through PDs and peers) offered them through this program. However, despite articulating a need for other resources and development, participation and feedback were inconsistent. We must continue to have difficult conversations and make hard decisions to determine if and how we are reaching the highest goal of programs like this: equity for caregivers in science and medicine. This includes asking ourselves who is eligible, how we determine that, and how we build out programming that will help them on their career paths without creating unwieldy requirements.

## Abbrevations used

DDCF: Doris Duke Charitable Foundation
DSA: Distinguished Scholars Award
FCRS: Fund to Retain Clinical Scientists
ISMMS: Icahn School of Medicine at Mount Sinai
NIH: National Institutes of Health
OCOS: Office of Career Opportunity and Support
PDs: program directors
STEMM: Science, technology, engineering, mathematics, and medicine
